# Unawareness of Hepatitis B Virus Infection confers on Higher Rate of Metabolic Syndrome: A Community-based Study

**DOI:** 10.1038/s41598-017-10029-2

**Published:** 2017-08-29

**Authors:** Cheng-Hung Chien, Li-Wei Chen, Chih-Lang Lin, Su-Wie Chang, Yu-Chiau Shyu, Kuan-Fu Chen, Shuo-Wei Chen, Ching-Chih Hu, Chia-Ying Yu, Rong-Nan Chien

**Affiliations:** 1 Liver Research Unit, Chang Gung Memorial Hospital, Keelung, Taiwan, R.O.C.; 2 Community Medicine Research Center, Keelung Chang Gung Memorial Hospital, Keelung, Taiwan, R.O.C.; 3 Clinical Informatics and Medical Statistics Research Center, College of Medicine, Chang Gung University, Taoyuan, Taiwan, R.O.C.; 4 Division of Allergy, Asthma, and Rheumatology, Department of Pediatrics, Chang Gung Memorial Hospital, Taoyuan, Taiwan, R.O.C.; 5 Institute of Molecular Biology, Academia Sinica, Nankang, Taipei, Taiwan, R.O.C.; 6 Department of Nursing, Chang Gung University of Science and Technology, Guishan, Taiwan, R.O.C.; 7 Department of Emergency Medicine, Chang Gung Memorial Hospital, Keelung, Taiwan, R.O.C.

## Abstract

The objective of this study was to determine whether awareness of hepatitis B virus (HBV) serostatus was discordant with metabolic syndrome (MetS) among people with chronic HBV infection. We conducted a community-based study in four Taiwanese districts. A total of 3493 adult participants were recruited. Participants who were hepatitis B surface antigen (HBsAg) seropositive and had self-reported HBV infection were considered aware of hepatitis B (aHB); those who denied a history of HBV infection were considered unaware of hepatitis B (uaHB). Among the 454 participants who were HBsAg seropositive, 275 (60.6%) were aHB and 179 (39.3%) were uaHB. Hypertriglyceridemia showed significant inverse association with HBsAg seropositive, especially among those who were aHB. Insulin resistance was significantly, positively associated with HBsAg seropositive, especially among participants who were uaHB. Those who were uaHB had a higher risk of central obesity, hyperglycemia, insulin resistance, and MetS than those who were aHB (odds ratio = 2.33, 1.64, 2.15, 1.85, respectively, all p < 0.05). The association among the prevalence of MetS, its individual components and HBsAg seropositivity varies according to awareness of HBV infection. It is important to recognize an individual’s risk for MetS, especially who were unaware of HBV infection.

## Introduction

The hepatitis B virus (HBV) and its relationship with the metabolic syndrome (MetS) have recently become the focus of research^1^. Many, but not all, studies indicated that patients with chronic hepatitis B (CHB) are at a lower risk of MetS, non-alcohol fatty liver disease, and dyslipidemia^2^. Unlike the hepatitis C virus (HCV), the causal relationship between HBV and the development of metabolic disorder is not straightforward. HBV carriers are at a much higher risk of developing cirrhosis and hepatocellular carcinoma, which causes death attributed to the liver disease. According to the Health Belief Model, risk perception is the primary motive to change behavior, and the greater the perceived threat, the more likely it is that an individual will change his or her behavior^3^. Shin *et al*. found that HBV carriers who were aware of their serostatus had a favorable behavior change compared with those who were not aware of their status^4^. People who are more aware and knowledgeable of their HBV serostatus tend to pay more attention to their health status. Similarly, an individual’s knowledge, attitudes, and behavior play significant roles in prevention and management of the risk factors for MetS^5^. Recognizing their personal risk and making behavior and lifestyle changes may reduce the prevalence of conditions relevant to MetS.

Little information about the impact of awareness of HBV serostatus on perceived health status and health-promoting behaviors is available in the literature. Previous studies analyzing the association between HBV infection and MetS focused on different source populations, including people undergoing health examinations^6, 7^, community-based samples^8^, population-based samples^9^, and a subset of national samples^10^. The prevalence of awareness of HBV serostatus were not reported in these studies. The awareness of HBV serostatus may vary considerably among different samples, depending on the age, education level, urban residence, and family history of liver disease^11^. More importantly, when the relationship between chronic HBV infection and MetS is studied, a behavioral mechanism, in addition to the potential biological mechanism, should be examined. Hence, the objective of this community-based study was to determine whether awareness of HBV serostatus was discordant with MetS and its individual component among people with CHB.

## Results

After excluding participants who were antibody to HCV (anti-HCV) positive (n = 143), had a history of cancer (n = 125), or had incomplete data on the MetS survey (n = 17), hepatitis B surface antigen (HBsAg) or anti-HCV tests (n = 10), or did not answer to the question of HBV infection (n = 23), 3,493 participants were included in the analysis. The prevalence of MetS was 31.6%. Participants with MetS were older, had less formal education, exercised less, and were more likely to live in a rural area, smoke, and alcohol consumption than those without MetS (Table 1). Among the participants with MetS, 14% had elevated aspartate aminotransferase (AST), 22% had elevated alanine aminotransferase (ALT), 39.2% had elevated γ-glutamyltranspeptidase (GGT), and 78.3% had fatty liver. Participants with MetS were more likely to have significant fibrosis (AST-to-platelet ratio index (APRI) > 0.5) and advanced fibrosis (APRI > 1.0) of the liver compared with those without MetS (11.8% vs 6.4%, p < 0.001; and 1.5% vs. 0.7%, p = 0.036, respectively). The area under receiver operating characteristic curves of homeostatic model assessment - insulin resistance (HOMA-IR) for MetS diagnosis was 0.797(95% confidence interval: 0.781–0.812). The optimal cut-off for the diagnosis of MetS was 1.905 (sensitivity: 68.6%, specificity: 77.3%). IR was defined as HOMA-IR ≥ 1.9.

**Table 1:** Demographic and clinical characteristics of participants with and without metabolic syndrome.

	No Metabolic syndrome	Metabolic syndrome	p
Number	2390	1103	
Male (%)	846(35.4)	403(36.5)	0.514
Age, years, mean (SD)	55.4 ± 13.2	61.2 ± 12.0	<0.001
BMI, kg/m^2^ (%)			
<24,	1385(58.0)	173(15.8)	<0.001
24–27	654(27.4)	354(32.3)	
≥27	347(14.5)	570(52.0)	
Live in rural area (%)	840(35.1)	471(42.7)	<0.001
AST > 34 U/L (%)	159(6.7)	156(14.1)	<0.001
ALT > 36 U/L (%)	211(8.8)	245(22.2)	<0.001
GGT > 26 U/L (%)	462(19.3)	432(39.2)	<0.001
HBsAg(+) (%)	319(13.3)	135(12.2)	0.365
Report a history of HBV infection (%)	256(10.7)	83(7.5)	0.003
Smoking (%)			
Never	1811(78.9)	803(75.8)	0.016
<20 pack year	271(11.8)	125(11.8)	
≥20 pack year	212(9.2)	132(12.5)	
Alcohol consumption (%)			
Non drinker	1561(65.9)	752(68.8)	0.016
Normal drinker	546(23.1)	206(18.8)	
Heavy drinker	260(11.0)	135(12.4)	
Fatty liver (%)			
None	384(51.6)	70(21.7)	<0.001
Mild	245(32.9)	72(22.4)	
Moderate	105(14.1)	139(43.2)	
Severe	10(1.3)	41(12.7)	
Family history of HTN (%)	891(37.3)	424(38.4)	0.511
Family history of DM (%)	550(23.0)	286(25.9)	0.06
Educational level (%)			
No education completed	153(6.5)	149(13.7)	<0.001
Elementary school	518(21.8)	346(31.7)	
Middle school	382(16.1)	173(15.9)	
High school	713(30.1)	265(24.3)	
College and higher	605(25.5)	157(14.4)	
Exercise (%)			
0–30 minutes/day	826(35.6)	375(35.1)	0.001
30–60 minutes/day	986(42.5)	513(48.1)	
>60 minutes/day	508(21.9)	179(16.8)	
HOMA-IR, mean (SD)	1.58 ± 1.54	3.79 ± 5.14	<0.001
APRI > 0.5 (%)	153(6.4)	130(11.8)	<0.001
APRI > 1.0 (%)	17(0.7)	16(1.5)	0.036

BMI: body mass index; AST: aspartate aminotransferase; ALT: alanine aminotransferase; GGT: gamma glutamyltransferase; HBsAg: hepatitis B surface antigen; HBV: hepatitis B virus; HOMA-IR: homeostatic model assessment- insulin resistance; APRI: aspartate aminotransferase-to-platelet ratio index.

Among the 454 participants who were HBsAg seropositive, 275 (60.6%) were aware of hepatitis B (aHB) and 179 (39.3%) were unaware of hepatitis B (uaHB). The proportion who were HBsAg positive did not differ between participants with and without MetS (12.2% vs. 13.3%, p = 0.563). The proportion who self-reported a history of HBV infection was lower in participants with MetS than in those without it (7.5% vs. 10.7%, p = 0.003). MetS was not related to APRI level among HBsAg seropositive participants. Compared with participants who were aHB, those who were uaHB were older, more likely to live in a rural area, had less formal education, and were less likely to have a family history of liver disease (Table 2).

**Table 2:** Comparison of characteristics of HBsAg seropositive participants with and without awareness of hepatitis B virus infection

	Unaware of hepatitis B	Aware of hepatitis B	P value
Number	179	275	
Male (%)	64(35.8)	108(39.3)	0.450
Age, years, mean (SD)	59.3 ± 12.7	53.8 ± 11.6	<0.001
Age, years (%)			
30–49	41(22.9)	92(33.5)	0.001
50–64	79(44.1)	131(47.6)	
>65	59(33.0)	52(18.9)	
BMI, kg/m^2^, mean (SD)	25.3 ± 4.0	24.3 ± 3.6	0.004
<24 (%)	74(42.0)	130(47.4)	0.076
24–27 (%)	51(29.0)	90(32.8)	
≥27 (%)	51(29.0)	54(19.7)	
Live in rural area (%)	97(54.2)	91(33.2)	<0.001
Education level (%)			
No education completed	26(14.9)	8(2.9)	<0.001
Elementary school	56(32.2)	59(21.7)	
Middle school	34(19.5)	44(16.2)	
High school	38(21.8)	81(29.8)	
College and higher	20(11.5)	80(29.4)	
Marry status (%)	167	267	
Never married	14(8.4)	21(7.9)	0.203
Married	127127(76.0)	222(83.1)	
Divorce or separate	11(6.6)	11(4.1)	
Married without spouse	15(9.0)	13(4.9)	
Exercise (%)			
0–30 minutes/day	67(39.6)	87(32.7)	0.268
30–60 minutes/day	66(39.1)	123(46.2)	
>60 minutes/day	36(21.3)	56(21.1)	
Alcohol consumption (%)			
Non drinker	129(75.4)	180(65.7)	0.066
Regular drinker	22(12.9)	57(20.8)	
Heavy drinker	20(11.7)	37(13.5)	
Smoking (%)			
Never	124(73.8)	199(75.4)	0.729
<20 pack-year	20(11.9)	34(12.9)	
≥20 pack -year	24(14.3)	13(11.7)	
Father has liver disease (%)	7(3.9)	28(10.2)	0.014
Mother has liver disease (%)	4(2.2)	26(9.5)	0.002
Sibling has liver disease (%)	4(2.2)	24(8.7)	0.005

BMI: body mass index. SD: standard deviation.

### Relationship between HBV and Metabolic Syndrome

Among the whole sample (n = 3493), the prevalence of MetS was lower among those who were HBsAg seropositive compared with those who were seronegative, but the difference was not statistically significant (29.7% vs. 31.9%, p = 0.366) (Tables 3 and 4). HBsAg seropositivity was inversely associated with hypertriglyceridemia (aOR = 0.66, 95% CI 0.51–0.87) and positively related to HOMA-IR ≥ 1.9 (aOR = 1.50, 95% CI 1.19–1.90), after adjusting for age, sex, body mass index (BMI), smoking, alcohol consumption, and exercise. HBsAg seropositivity was not significantly associated with elevated blood pressure, hypertriglyceridemia, low low-density lipoprotein cholesterol (LDL-C) level, hyperglycemia, hypercholesterolemia, or fatty liver.

**Table 3:** Clinical profiles of participants by HBsAg positivity and awareness of serostatus

	HBsAg(−)	HBsAg(+)	uaHB	aHB
Number	3039	454	179	275
BMI < 24 kg/m^2^ (%)	1354(44.6)	204(45.3)	74(42.0)	130(47.4)
BMI > 24, <27 kg/m^2^ (%)	867(28.6)	141(31.3)	51(29.0)	90(32.8)
BMI ≥ 27 kg/m^2^ (%)	812(26.8)	105(23.3)	51(25.0)	54(19.7)
Metabolic syndrome (%)	968(31.9)	135(29.7)	72(40.2)	63(22.9)
Elevated blood pressure (%)	1756(57.8)	262(57.7)	115(64.2)	147(53.5)
Central obesity (%)	1238(40.7)	175(38.5)	94(52.5)	81(29.5)
Hypertriglyceridemia (%)	797(26.2)	86(18.9)	42(23.5)	44(16.0)
Low HDL-C (%)	660(21.7)	98(21.6)	45(25.1)	53(19.3)
Hyperglycemia (%)	1220(40.1)	161(35.5)	82(45.8)	79(28.7)
Total cholesterol ≥ 200 mg/dL (%)	1790(58.9)	247(54.4)	95(53.1)	152(55.3)
LDL-C ≥ 130 mg/dL (%)	1305(42.9)	185(40.7)	71(39.7)	114(41.5)
HOMA-IR ≥ 1.9 (%)	1124(37.0)	188(41.4)	95(53.1)	93(33.8)
Fatty liver (%)	532/910(58.5)	82(52.6)	27/49(55.1)	55/107(51.4)
APRI > 0.5 (%)	220(7.2)	63(13.9)	28(15.6)	35(12.7)
APRI > 1.0 (%)	25(0.8)	8(1.8)	5(2.8)	3(1.1)

HBsAg: hepatitis B surface antigen; uaHB: unaware of hepatitis B; aHB: aware of hepatitis B; BMI: body mass index; HDL-C: high-density lipoprotein cholesterol; LDL-C: low-density lipoprotein cholesterol; HOMA-IR: homeostatic model assessment- insulin resistance; APRI: aspartate aminotransferase-to-platelet ratio index.

**Table 4:** Comparison of clinical profiles of participants by HBsAg positivity and awareness of serostatus.

	HBsAg(+) vs HBsAg(−)				uaHB vs HBsAg(−)				aHB vs HBsAg(−)			
	OR (95% CI)	P-value	aOR (95% CI)	P-value	OR (95% CI)	P-value	aOR (95% CI)	P-value	OR (95% CI)	P-value	aOR (95% CI)	P-value
Metabolic syndrome	0.91(0.73–1.12)	0.366	1.05(0.81–1.36)	0.719	1.44(1.06–1.96)	0.021	1.42(0.97–2.07)	0.068	0.636(0.48–0.85)	0.002	0.83(0.59–1.17)	0.297
Elevated blood pressure	1.00(0.82–1.23)	0.977	1.12(0.89–1.52)	0.335	1.30(0.95–1.79)	0.098	1.18(0.81–1.72)	0.380	0.839(0.66–1.08)	0.165	1.08(0.81–1.45)	0.582
Central obesity	0.91(0.75–1.12)	0.912	1.03(0.77–1.38)	0.828	1.60(1.19–2.17)	0.002	1.73(1.11–2.69)	0.016	0.607(0.46–0.80)	<0.001	0.72(0.50–1.05)	0.091
Hypertriglyceridemia	0.66(0.51–0.84)	0.001	0.66(0.51–0.87)	0.003	0.86(0.60–1.23)	0.404	0.76(0.51–1.13)	0.179	0.54(0.38–0.75)	<0.001	0.60(0.42–0.85)	0.004
Low HDL-C	0.99(0.78–1.26)	0.949	0.86(0.63–1.18)	0.494	1.22(0.86–1.72)	0.273	1.25(0.86–1.83)	0.244	0.86(0.63–1.18)	0.345	0.95(0.68–1.32)	0.759
Hyperglycemia	0.82(0.67–1.01)	0.057	0.87(0.69–1.09)	0.230	1.26(0.93–1.71)	0.131	1.19(0.84–1.68)	0.326	0.60(0.46–0.79)	<0.001	0.70(0.52–0.94)	0.019
Total cholesterol ≥ 200 mg/dL	0.83(0.68–1.02)	0.070	0.87(0.71–1.08)	0.204	0.78(0.58–1.06)	0.107	0.79(0.57–1.09)	0.152	0.86(0.67–1.11)	0.24	0.93(0.72–1.20)	0.572
LDL-C ≥ 130 mg/dL	0.91(0.75–1.12)	0.378	0.94(0.75–1.15)	0.494	0.87(0.64–1.18)	0.355	0.82(0.59–1.14)	0.243	0.94(0.73–1.21)	0.633	1.00(0.77–1.30)	0.978
HOMA-IR ≥ 1.9	1.20(0.99–1.47)	0.071	1.50(1.19–1.90)	0.001	1.93(1.43–2.61)	<0.001	2.50(1.74–3.59)	<0.001	0.87(0.67–1.13)	0.295	1.08(0.8–1.46)	0.605
Fatty liver	0.79(0.56–1.11)	0.169	0.93(0.63–1.36)	0.692	0.88(0.50–1.58)	0.674	0.92(0.46–1.85)	0.823	0.75(0.50–1.12)	0.163	0.94(0.60–1.47)	0.780

Data are presented as odds ratio (95% confidence interval).

HBsAg: hepatitis B surface antigen; uaHB: unaware of hepatitis B; aHB: aware of hepatitis B; HDL-C: high-density lipoprotein cholesterol; LDL-C: low-density lipoprotein cholesterol; HOMA-IR: homeostatic model assessment- insulin resistance.

aOR: adjusted odds ratio. Adjusted for age, gender, BMI, smoking status, alcohol consumption, and exercise time.

Further analysis of association stratified by hepatitis B awareness status, participants who were uaHB were more likely to have MetS than HBsAg seronegative participants (40.2% vs. 31.9%, p = 0.021). However, the association became marginal significance after adjusting for the potential confounders (p = 0.068). Participants who were uaHB had a higher risk of central obesity and HOMA ≥ 1.9 than HBsAg seronegative participants (aOR = 1.73, 95% CI 1.11–2.69; and aOR = 2.00, 95% CI 1.37–2.89, respectively). Participants who were aHB were less likely to have MetS than HBsAg seronegative participants (22.9% vs. 31.9%, p = 0.002) but this was not statistically significant after adjusting for potential confounders (p = 0.297). aHB was inversely associated with hypertriglyceridemia and hyperglycemia (aOR = 0.6, 95% CI 0.42–0.85; and aOR = 0.7, 95% CI 0.52–0.94, respectively). Levels of total cholesterol, LDL-C, and HDL-C were not significantly associated with awareness of HBV serostatus.

### Comparison of HBV carriers who were and were not aware of their HBV serostatus

The relationship between HBV awareness and MetS among the HBsAg seropositive participants is shown in Table 5. After adjusting for age and sex, the aOR of having MetS was 1.85 (95% CI 1.20–2.83) in uaHB compared with aHB. When each metabolic component was analyzed separately, the aOR of having central obesity, hyperglycemia, and HOMA-IR ≥ 1.9 were significantly higher among uaHB compared with aHB participants (aOR = 2.33, 95% CI 1.56–3.49; aOR = 1.64, 95% CI 1.08–2.49; and aOR = 2.15, 95% CI 1.45–3.20, respectively). Being uaHB was not significantly associated with elevated blood pressure, hypertriglyceridemia, hypercholesterolemia, high LDL-C level, low high-density lipoprotein cholesterol (HDL-C) level, fatty liver, or elevated AST, ALT or APRI levels.

**Table 5:** Comparison of clinical profiles of HBsAg seropositive participants (unaware of hepatitis B versus aware of hepatitis B).

	Crude OR (95% CI)	P	Adjusted OR (95% CI)	P
BMI ≥ 24 kg/m^2^	1.24(0.85–1.82)	0.262	1.18(0.79–1.76)	0.413
BMI ≥ 27 kg/m^2^	1.66(1.07–2.58)	0.024	1.85(1.17–2.92)	0.009
Metabolic syndrome	2.26(1.50–3.41)	<0.001	1.85(1.20–2.83)	0.005
Elevated blood pressure	1.57(1.06–2.30)	0.023	1.20(0.79–1.83)	0.391
Central obesity	2.65(1.79–3.92)	<0.001	2.33(1.56–3.49)	<0.001
Hypertriglyceridemia	1.60(1.00–2.58)	0.048	1.60(0.99–2.60)	0.057
Low HDL-C	1.40(0.90–2.21)	0.139	1.33(0.83–2.11)	0.234
Hyperglycemia	2.10(1.42–3.11)	<0.001	1.64(1.08–2.49)	0.020
Total cholesterol (≥200 mg/dL)	0.92(0.63–1.34)	0.646	0.89(0.64–1.31)	0.558
LDL-C (≥130 mg/dL)	0.93(0.63–1.36)	0.705	0.93(0.63–1.38)	0.713
HOMA-IR ≥ 1.9	2.21(1.51–3.25)	<0.001	2.15(1.45–3.20)	<0.001
Fatty liver	1.16(0.59–2.29)	0.668	1.17(0.59–2.31)	0.657
AST > 34 U/L	1.17(0.68–2.01)	0.583	1.10(0.63–1.93)	0.733
ALT > 36 U/L	0.98(0.59–1.61)	0.927	1.12(0.68–1.88)	0.673
APRI > 0.5	1.27(0.74–2.18)	0.381	1.14(0.65–1.99)	0.645
APRI > 1.0	2.61(0.62–11.04)	0.194	2.57(0.58–11.29)	0.212

Data are presented as odds ratio (95% confidence interval).

HBsAg: hepatitis B surface antigen; uaHB: unaware of hepatitis B; aHB: aware of hepatitis B; HDL-C: high-density lipoprotein cholesterol; LDL-C: low-density lipoprotein cholesterol; HOMA-IR: homeostatic model assessment- insulin resistance; AST: aspartate aminotransferase; ALT: alanine aminotransferase; APRI: aspartate aminotransferase-to-platelet ratio index. aOR: adjusted odds ratio. Adjusted for age and gender.

## Discussion

In this community-based cross-sectional study, the association between MetS and awareness of HBV serostatus was examined. The prevalence of MetS was not associated with HBsAg seropositivity. The prevalence of MetS was lower among participants who were aHB and higher among participants who were uaHB compared with HBsAg seronegative participants. This difference may explain the conflicting results of published studies. It suggests that the complex relationship between metabolic disorder and CHB is explained by not only biomedical (e.g., genetics, biological process), but also by biosocial (e.g., sex, race/ethnicity, education) and psychosocial (e.g., physical activity, diet intake, smoking) variables.

A significant inverse association between HBsAg seropositivity and hypertriglyceridemia was observed in this study. This finding is consistent with previous studies conducted among different ethnic groups^6, 7, 9, 12–16^. A potential mechanism is inhibition of the secretion of apolipoprotein B^17^, an essential component for the formation of very-low-density lipoprotein (VLDL) and LDL-C, by HBV X protein. The increase in HBV X protein contributes to lower levels of VLDL, a triglyceride-rich particle, and a consequent reduction in the serum triglyceride level^18^. A similar association was evident between aHB, but not uaHB, and hypertriglyceridemia. HBV awareness has implications for lipid profile changes in HBV carriers, and its mechanism needs further exploration.

IR is the principal pathophysiological mechanism that leads to MetS. However, the effect of chronic HBV infection on human insulin sensitivity and hyperglycemia is not consistent in the literature. HBsAg seropositive patients had higher HOMA-IR levels than controls in a study from Korea^19^. However, no significant association between chronic HBV infection and the HOMA-IR level was reported in a study from Taiwan^20^. A recent meta-analysis of HBV infection and risk of type 2 diabetes mellitus (DM), revealed that HBV itself might not be pro-diabetic^21^. Another meta-analysis showed a significantly higher prevalence of DM in the HBV-infected group than in the control group in the Asia-Pacific region^22^. In our study, HBsAg seropositive participants, particularly those who were uaHB, had a higher risk of IR compared with controls. Interestingly, aHB was not related to IR, and those participants had an even lower risk of hyperglycemia than controls. When studying the relationship between chronic HBV infection and IR, we suggest the influence of HBV awareness should be considered.

To the best of our knowledge, the impact of awareness of HBV serostatus on the risk of MetS has never been previously studied. ATP III, the National Heart, Lung and Blood Institute and the American Heart Association identified specific underlying risk factors for MetS, including obesity, physical inactivity, atherogenic diet, cigarette smoking, and family history of premature coronary heart disease^23, 24^. Other well-defined factors beyond the clinical criteria that define MetS, such as patients’ knowledge, attitudes, and behaviors, may contribute to the development of MetS and diseases for which it is a predisposing condition^5^. Compared with participants who were aHB, those who were uaHB had a higher risk of obesity, hyperglycemia, IR, and MetS in our study. Although our cross sectional data cannot address the questions of causality, we suppose biosocial and psychosocial factors may explain potential pathogenic mechanisms. The level of education was not associated with MetS in the National Health and Nutrition Examination Survey (NHANES) cohort^25^. However, among African-American females and males, more formal education (at least high school graduation) was associated with reduced risk of MetS, compared with lower educational status^26^. Our study revealed that participants who were aHB had more formal education and were more likely to have a family history of liver disease. The educational level may influence dietary choices, physical activity, and stress levels. In theory, they would have better medical knowledge and pay more attention to their health status. This population was mostly likely to embrace lifestyle changes and achieve good medical compliance and adherence.

In addition to socioeconomic status, rural-urban differences may play a role in the prevalence of MetS in developing nations^27^, but these associations are not consistent. In Korea, the age and sex adjusted prevalence of MetS was higher in rural than in urban communities^28^. In the present study, participants living in rural areas were more likely to have MetS compared with those living in urban areas. This may be attributed to the difference in accessibility of medical services and public awareness. Rural residents are less likely than urban residents to obtain certain preventive healthcare services^29^. Furthermore, socioeconomically disadvantaged older people in rural areas face personal, community, and healthcare barriers that limit their access to primary care^30^. We found that participants who were uaHB, compared to those who were aHB, were older and more likely to live in a rural area. They have higher risk of obesity and MetS probably because they receive less medical information and use less medical resources to improve their health.

Analysis of the NHANES III showed that type II DM and IR are independent predictors of overall mortality among people with CHB^31^. Excess BMI and MetS is a significant risk factor for the development of cirrhosis, HCC and intrahepatic cholangiocarcinoma^32–34^. It is reasonable to predict that people who are uaHB have a higher risk of liver fibrosis, cirrhosis, and HCC. People who are uaHB are more likely to be diagnosed at a later stage and antiviral therapy is delayed. Active screening programs and increased disease awareness are vital for preventing CHB disease progression.

The present study has several limitations. First, the study examined cross-sectional data; therefore, we could not conclude that a causal relationship existed between awareness of HBV serostatus and lower risk of the metabolic disorder. We suggested that individuals’ perceived threat and knowledge of and attitude toward the disease, along with their accessibility to medical resources determine this causality. Our postulation needs further prospective and longitudinal follow up study to validate. Second, we did not evaluate the time period of hepatitis B awareness and relevant lifestyle changes, such dietary habits, in the present study. A comprehensive behavioral assessment and a longitudinal follow-up study would be required to clarify the etiology. Third, we did not measure hepatitis B e-antigen serostatus and hepatitis B viral load to analyze different groups’ metabolic profile. A dose-response relation between viral replication and metabolic disorder may help establish causality. Instead, we found that no association was observed between MetS and the degrees of liver fibrosis among HBV carriers.

In conclusion, this community-based study indicated that the association among the prevalence of MetS, its individual components and HBsAg seropositivity varies according to awareness of HBV infection. Hypertriglyceridemia showed significant inverse association with HBsAg seropositivity, especially among those who were aHB. IR was significantly, positively associated with HBsAg seropositivity, especially among participants who were uaHB. Moreover, those who were uaHB had a higher risk of obesity, hyperglycemia, IR, and MetS than those who were aHB. In addition to an urgent need to promote awareness of HBV and the treatment of eligible patients, it is important to recognize an individual’s risk for MetS and provide interventions with specific management strategies.

## Methods

### Study population

We conducted a community-based cross-sectional study from August 2013 to August 2015 in four Taiwanese districts (Wan-li, Gong-liao, Rul-fan, and An-le). Three districts were rural townships on the northeastern seaboard, and one was urban. Adult participants (≥30 years old) were recruited from the community by public service announcements, talks to community groups, and notices in clinics. Written informed consent was obtained from each of the participants. The study was conformed to the ethical guidelines of the Declaration of Helsinki, and was performed with the approval of the ethical committee of the Keelung Chang Gung Memorial Hospital.

### Clinical evaluation and laboratory tests

The data related to alcohol consumption, smoking history, physical activity, family history, and medical history were obtained by a structured in-person interview that was administered by trained nurses, research assistants, and medical students. Heavy alcohol consumption was defined as alcohol intake >30 g daily in males and >20 g daily in females^35^. Normal consumption (light to moderate alcohol intake) was defined as a history of alcohol consumption but no heavy alcohol consumption. Blood pressure, height, weight, and waist circumference were measured with the subjects wearing light clothes and no shoes. BMI was calculated as weight in kilograms divided by the square of the height in meters. The BMI cutoffs suggested by the Department of Health in Taiwan were used. These cutoffs were used to create normal (18.5 ≤ BMI < 24), overweight (24 ≤ BMI < 27) and obese (BMI ≥ 27) categories. All participants were asked to fast overnight (≥8 hours) before blood sample collection. Blood tests included liver biochemistry, glucose, lipid profile, HBsAg, and anti-HCV. The APRI was used to assess liver fibrosis. The APRI was calculated as follows:

$${\rm{APRI}}={\rm{AST}}(/{\rm{upper}}\,{\rm{limit}}\,{\rm{of}}\,{\rm{normal}})\times 100/{\rm{platelet}}\,{\rm{count}}({10}^{9}/{\rm{L}})$$

APRI thresholds of 0.5 and 1.0 resulted in sensitivity and specificity values of 70.0% and 60.0%, 50.0% and 83.0% for significant fibrosis and advanced fibrosis, respectively^36^. Since IR is one of the key mechanisms for MetS development, we assessed IR using the HOMA-IR score^37^. The HOMA-IR score was calculated by the following formula:

$$\begin{array}{c}\mathrm{HOMA}\, \mbox{-} \,\mathrm{IR}={\rm{Fasting}}\,{\rm{plasma}}\,{\rm{insulin}}\,(m{\rm{U}}/{\rm{L}})\\ \quad \quad \quad \quad \quad \,\times {\rm{fasting}}\,{\rm{plasma}}\,{\rm{glucose}}\,({\rm{mmol}}/{\rm{L}})/22.5\end{array}$$

Some communities were selected for hepatic ultrasonography examination based on the availability of public facilities. An ultrasonography scoring system, which included assessment of the liver surface, liver parenchyma, hepatic vessels, and spleen size, was developed to evaluate the degree of hepatic fibrosis^38^. The degree of hepatic steatosis was graded as none, mild, moderate, or severe based on the discrepancy of echogenicity between the liver and kidneys, the degree of posterior attenuation, and the visibility of the vessels^39^. After ultrasonography, the presence of cirrhosis and/or fatty liver was recorded. A total of 1,066 (30.5%) participants received abdominal ultrasonography.

### Hepatitis B awareness and metabolic syndrome

All participants were asked whether they had ever been infected with HBV. Participants who were HBsAg seropositive and had self-reported HBV infection were considered aHB. Participants who were HBsAg seropositive and denied a history of HBV infection were considered uaHB.

A race-specific waist circumference threshold, based on the National Cholesterol Education Program Adult Treatment Panel III (NCEP ATP III) criteria^23, 40, 41^ was utilized to prevent distortions in MetS prevalence. According to the ATP III criteria, MetS was defined as the presence of at least three of the following five traits: central obesity (based on the Asian waist circumference cut-offs, males: >90 cm, females: >80 cm); blood pressure ≥130/85  mm Hg or drug treatment for essential hypertension; serum HDL-C level <40 mg/dL in males or <50 mg/dL in females or drug treatment for low HDL-C; serum triglycerides level ≥150 mg/dL or drug treatment for elevated triglycerides; and fasting plasma glucose level ≥100 mg/dL or drug treatment for DM.

### Statistical analysis

Continuous variables were expressed as mean ± standard deviation. Statistical comparisons between groups of patients were performed using the t-test for continuous variables and Pearson chi-square test for categorical variables. Receiver operating characteristics (ROC) analysis with maximization of the Youden index (sensitivity + specificity  −  1) was used to establish the optimal cut-off for HOMA-IR to predict MetS. We conducted unadjusted and multivariate adjusted logistic regression analyses to determine if HBV awareness was associated with odds of MetS and its individual components. Database manipulation and analyses were performed using SPSS, version 19 (SPSS Inc., Chicago, IL, USA). The odds ratio (OR), adjusted OR (aOR), 95% confidence interval (CI), and p-values were calculated. A p-value < 0.05 was considered statistically significant. The datasets generated during and/or analysed during the current study are available from the corresponding author on reasonable request.

## References

[1] Chiang CH, Huang KC (2014). Association between metabolic factors and chronic hepatitis B virus infection. World J Gastroenterol..

[2] Jarcuska P, Drazilova S, Fedacko J, Pella D, Janicko M (2016). Association between hepatitis B and metabolic syndrome: Current state of the art. World J Gastroenterol..

[3] Janz NK, Becker MH (1984). The Health Belief Model: a decade later. Health Educ Q..

[4] Shin A (2009). Factors associated with awareness of infection status among chronic hepatitis B and C carriers in Korea. Cancer Epidemiol Biomarkers Prev..

[5] Lewis SJ, Rodbard HW, Fox KM, Grandy S, Group SS (2008). Self-reported prevalence and awareness of metabolic syndrome: findings from SHIELD. Int J Clin Pract..

[6] Chung TH, Kim MC, Kim CS (2014). Association between Hepatitis B Surface Antigen Seropositivity and Metabolic Syndrome. Korean J Fam Med..

[7] Li WC (2013). Association between the hepatitis B and C viruses and metabolic diseases in patients stratified by age. Liver int..

[8] Huang CY (2016). Relationship between chronic hepatitis B and metabolic syndrome: A structural equation modeling approach. Obesity..

[9] Jan CF (2006). A population-based study investigating the association between metabolic syndrome and hepatitis B/C infection (Keelung Community-based Integrated Screening study No. 10). Int J Obesity..

[10] Jinjuvadia R, Liangpunsakul S (2014). Association between metabolic syndrome and its individual components with viral hepatitis B. Am J Med Sci..

[11] Chien C. H. *et al*. Seroprevalence and awareness of viral hepatitis in northeastern coast region of Taiwan – A community-based study. Proceeding of Asian Pacific Association for the Study of the Liver single topic conference on hepatitis C. *June* 10–12, Poster 151 (2016).

[12] Luo B, Wang Y, Wang K (2007). Association of metabolic syndrome and hepatitis B infection in a Chinese population. Clinica chimica acta..

[13] Chen JY (2010). Lower prevalence of hypercholesterolemia and hyperglyceridemia found in subjects with seropositivity for both hepatitis B and C strains independently. J Gastroenterol Hepatol..

[14] Wong VW (2012). Hepatitis B virus infection and fatty liver in the general population. J Hepatol..

[15] Hsu CS (2012). Impact of hepatitis B virus infection on metabolic profiles and modifying factors. J Viral Hepat..

[16] Choi JS (2015). Serum HBV surface antigen positivity is associated with low prevalence of metabolic syndrome in Korean adult men. J Epidemiol..

[17] Kang SK (2004). The hepatitis B virus X protein inhibits secretion of apolipoprotein B by enhancing the expression of N-acetylglucosaminyltransferase III. J Biol Chem..

[18] Chiang CH (2013). Association between obesity, hypertriglyceridemia and low hepatitis B viral load. Int J Obes..

[19] Lee JG (2012). Association of chronic viral hepatitis B with insulin resistance. World J Gastroenterol..

[20] Wang CC, Hsu CS, Liu CJ, Kao JH, Chen DS (2008). Association of chronic hepatitis B virus infection with insulin resistance and hepatic steatosis. J gastroenterol hepatol..

[21] Zhang J, Shen Y, Cai H, Liu YM, Qin G (2015). Hepatitis B virus infection status and risk of type 2 diabetes mellitus: A meta-analysis. Hepatol Res..

[22] Cai C (2015). Association between hepatitis B virus infection and diabetes mellitus: A meta-analysis. Exp Ther Med..

[23] Expert Panel on Detection E, Treatment of High Blood Cholesterol in A. Executive Summary of The Third Report of The National Cholesterol Education Program (NCEP) Expert Panel on Detection, Evaluation, And Treatment of High Blood Cholesterol In Adults (Adult Treatment Panel III). *JAMA*. **285**, 2486–2497 (2001). 10.1001/jama.285.19.2486 11368702

[24] Grundy S. M. *et al*. Definition of metabolic syndrome: Report of the National Heart, Lung, and Blood Institute/American Heart Association conference on scientific issues related to definition. *Circulation*. **109**, 433–438 (2004). 10.1161/01.CIR.0000111245.75752.C6 14744958

[25] Park YW (2003). The metabolic syndrome: prevalence and associated risk factor findings in the US population from the Third National Health and Nutrition Examination Survey, 1988–1994. Arch Intern Med..

[26] Lucove JC, Kaufman JS, James SA (2007). Association between adult and childhood socioeconomic status and prevalence of the metabolic syndrome in African Americans: the Pitt County Study. Am J Public Health..

[27] Cornier MA (2008). The metabolic syndrome. Endocr Rev..

[28] Lim S (2006). A rural-urban comparison of the characteristics of the metabolic syndrome by gender in Korea: the Korean Health and Genome Study (KHGS). J Endocrinol Invest..

[29] Casey MM, Thiede Call K, Klingner JM (2001). Are rural residents less likely to obtain recommended preventive healthcare services?. Am J Prev Med..

[30] Ford JA, Wong G, Jones AP, Steel N (2016). Access to primary care for socioeconomically disadvantaged older people in rural areas: a realist review. BMJ Open..

[31] Stepanova M, Rafiq N, Younossi ZM (2010). Components of metabolic syndrome are independent predictors of mortality in patients with chronic liver disease: a population-based study. Gut..

[32] Yu MW (2008). Body-mass index and progression of hepatitis B: a population-based cohort study in men. J Clin Oncol..

[33] Wong GL (2009). Metabolic syndrome increases the risk of liver cirrhosis in chronic hepatitis B. Gut..

[34] Welzel TM (2011). Metabolic syndrome increases the risk of primary liver cancer in the United States: a study in the SEER-Medicare database. Hepatology..

[35] Farrell GC, Chitturi S, Lau GK, Sollano JD (2007). Asia-Pacific Working Party on NAFLD. Guidelines for the assessment and management of non-alcoholic fatty liver disease in the Asia-Pacific region: executive summary. J Gastroenterol Hepatol..

[36] Xiao G, Yang J, Yan L (2015). Comparison of diagnostic accuracy of aspartate aminotransferase to platelet ratio index and fibrosis-4 index for detecting liver fibrosis in adult patients with chronic hepatitis B virus infection: a systemic review and meta-analysis. Hepatology..

[37] Ferrannini E, Haffner SM, Mitchell BD, Stern MP (1991). Hyperinsulinaemia: the key feature of a cardiovascular and metabolic syndrome. Diabetologia..

[38] Hung CH (2003). Correlation between ultrasonographic and pathologic diagnoses of hepatitis B and C virus-related cirrhosis. J Gastroenterol..

[39] Lin D (1993). Clinical significance of ultrasonographic fatty liver in asymptomatics: analysis of 1040 check-up subjects. J Med ultrasound..

[40] Alberti KG, Zimmet P, Shaw J (2006). Metabolic syndrome–a new world-wide definition. A Consensus Statement from the International Diabetes Federation. Diabet Med..

[41] Grundy SM (2005). Diagnosis and management of the metabolic syndrome: an American Heart Association/National Heart, Lung, and Blood Institute Scientific Statement. Circulation..

